# An Overview of Registered Clinical Trials on Glucosinolates and Human Health: The Current Situation

**DOI:** 10.3389/fnut.2021.730906

**Published:** 2021-10-27

**Authors:** Mirko Marino, Daniela Martini, Samuele Venturi, Massimiliano Tucci, Marisa Porrini, Patrizia Riso, Cristian Del Bo'

**Affiliations:** Department of Food, Environmental and Nutritional Sciences (DeFENS), Università degli Studi di Milano, Milan, Italy

**Keywords:** bioactives, Brassicaceae, sulfur compounds, clinical trials, human nutrition, health outcomes, food, food extracts

## Abstract

Epidemiological studies suggest a potential role of glucosinolates (GSLs) and isothiocyanates on human health. However, evidence from intervention studies, due to heterogeneity in features of study design, duration, participants, food or food components administered, and outcomes analyzed, is still insufficient. The current review aims to provide an overview of the trials on GSLs and GSL-rich foods registered over the last 20 years with the intention to summarize the main topics and results, but also the existing gaps that still need to be covered. Studies were collected by using ClinicalTrials.gov and the International Standard Randomized Controlled Trial Number (ISRCTN) registry. A total of 87 registered trials were identified with which most of them were performed by using extracts or pure compounds (*n* = 60) while few were conducted with GSL-rich foods (*n* = 27). In detail, sulforaphane was the most investigated compound, while broccoli was the most frequent food tested in the trials. The majority of the studies assessed the health effects of GSLs focusing on outcomes related to cancer and cognitive function, even if the current findings are not univocal. Emerging topics also included the study of GSLs and gut microbiota interaction and impact on skin health. Further attention was also drawn to the bioavailability of GSLs and/or derivatives from foods, extracts, and single compounds by also considering the contribution of the different genetic polymorphisms. In conclusion, although considerable efforts have been made to study GSLs and GSL-rich foods, further studies are necessary to provide evidence-based research and to corroborate the findings obtained. The interindividual response due to genetic polymorphisms should be further investigated in order to explore the contribution to the overall beneficial effect.

## Introduction

Glucosinolates are a large group of plant secondary metabolites containing sulfur groups that are mainly found in cruciferous plants, such as broccoli, cauliflower, Brussel sprouts, cabbage, kale, watercress, and bok choy in which they play an important role in the defense mechanisms (1). From a chemical point of view, glucosinolates (GSLs) are composed of a thiohydroximate-O-sulfonate group linked to glucose, and an alkyl, aralkyl, and orindolyl side chain (2). The side-chain of the O-sulfate thiohydroximate moiety contributes to the diversity of natural GSLs, with more than 130 structures discovered so far (3–5).

Glucosinolates are stable in plant cells, but when the plant is damaged such as in the case of cutting or chewing, they can be degraded by the enzyme myrosinase (6, 7). In detail, two different types of this enzyme exist: plant myrosinase, which coexists with GSLs in plants although physically segregated in vacuoles and thus not directly in contact with them, and bacterial myrosinase, which mainly acts in the colon (5). The thermal inactivation of myrosinase preserves some intact GSLs in cooked vegetables, and steaming was reported as the best cooking method to preserve them (8). The activity of myrosinase results in the production of a wide range of breakdown products, among which isothiocyanates (ITCs) are the most abundant (2).

The abundance of GSLs in Brassica vegetables makes these compounds of interest in the nutritional research field. Thus, the health benefits of GSLs and their breakdown products have been largely studied in the last decades in addition to considering the impact of several food-related (e.g., variety, agronomic factors, storage, and processing of the vegetables prior to consumption) and human-related (e.g., genetic factors, age, smoking habits) factors (9–16). These variables can affect the levels of GSLs ingested, their bioavailability and metabolism, and, in turn, their potential role on human health (17–19).

The study of the bioavailability of GSLs and derivatives is quite complex and, only recently, studies have focused their attention on understanding the absorption and metabolism of GSLs and breakdown products. The currently available data are derived mainly from the post-absorptive metabolism of ITCs reporting a urinary excretion of conjugated ITC metabolites following the intake of different cruciferous cooked vegetables (20–22). It has been documented that when Brassica vegetables are consumed without processing, plant myrosinase is able to hydrolyze GSLs in the proximal part of the gastrointestinal tract into different metabolites (e.g., ITCs, nitriles, oxazolidine-2-thiones, and indole-3-carbinols), while when plants are thermally treated before consumption, myrosinase is inactivated and GSLs move into the colon where they are extensively hydrolyzed by bacteria and then absorbed and/or excreted (2). An *in vivo* study conducted in rats by using radiolabeled ITCs documented a rapid absorption (at about 3 h from the intake) of ITCs (23). Another study documented that the urinary concentration of native GSLs may reach up to 5% of the ingested dose followed by conjugation to glutathione at a hepatic level and then a urinary excretion in the form of mercapturic acid accounting for 12–80% of the ingested dose of ITCs (24). Regarding the impact of microbiota, few studies have been performed evaluating the contribution on GSLs metabolism and the impact of GSLs on microbiota composition. Data from *in vitro* studies reported the formation of amines and nitriles from GSLs (23, 25).

Regarding the contribution of GSLs and GSL-rich foods on human health, epidemiological evidence seems to support their role against the onset of non-communicable diseases, particularly those related but not limited to cancer (26–28). Cohort studies suggest an inverse association between the intake of broccoli and the risk of all cancers taken together (29) and an inverse association between consumption of Brassica vegetables and the risk of stomach cancer (30). A consistent inverse association between the risk of lung, stomach, colon, and rectal cancer and the high consumption of Brassica vegetables has been shown by case-control studies (31–33).

With the intention to investigate the main topics of research in the GSLs field, the aim of the present study was to perform an overview of the registered clinical trials in the last 20 years with the purpose of summarizing the main topics, results, and the existing gaps that still need to be covered. The information was obtained by examining public registries and available publications.

## Materials and Methods

### Search Strategy and Data Selection

The study was conducted by searching clinical trials registered in “ClinicalTrials.gov” (34) and International Standard Randomized Controlled Trial Number (ISRCTN) registry (35). The search was firstly conducted on June 30, 2020, and updated on April 30, 2021, to identify additional studies. The search strategies involved the combination of different terms, using a syntax that was adapted for each registry. Below, the search strategy used for each registry is given.

- ClinicalTrials.gov: glucosinolates OR isothiocyanates OR glucoraphanin OR sulforaphane OR glucobrassicin OR 3-indolylmethyl OR indol-3-carbinol OR thioglycosides OR thioglucosides OR progoitrin OR goitrin OR l-5-vinyl-2-thioöxazolidine OR sinagrin OR 4-methylsulfinylbutyl.
- ISRCTN Registry: (glucosinolates) OR (isothiocyanates) OR (glucoraphanin) OR (sulforaphane) OR (glucobrassicin) OR (3-indolylmethyl) OR (indol-3-carbinol) OR (thioglycosides) OR (thioglucosides) OR (progoitrin) OR (goitrin) OR (l-5-vinyl-2-thioöxazolidine) OR (sinagrin) OR (4-methylsulfinylbutyl).

**Figure 1:**
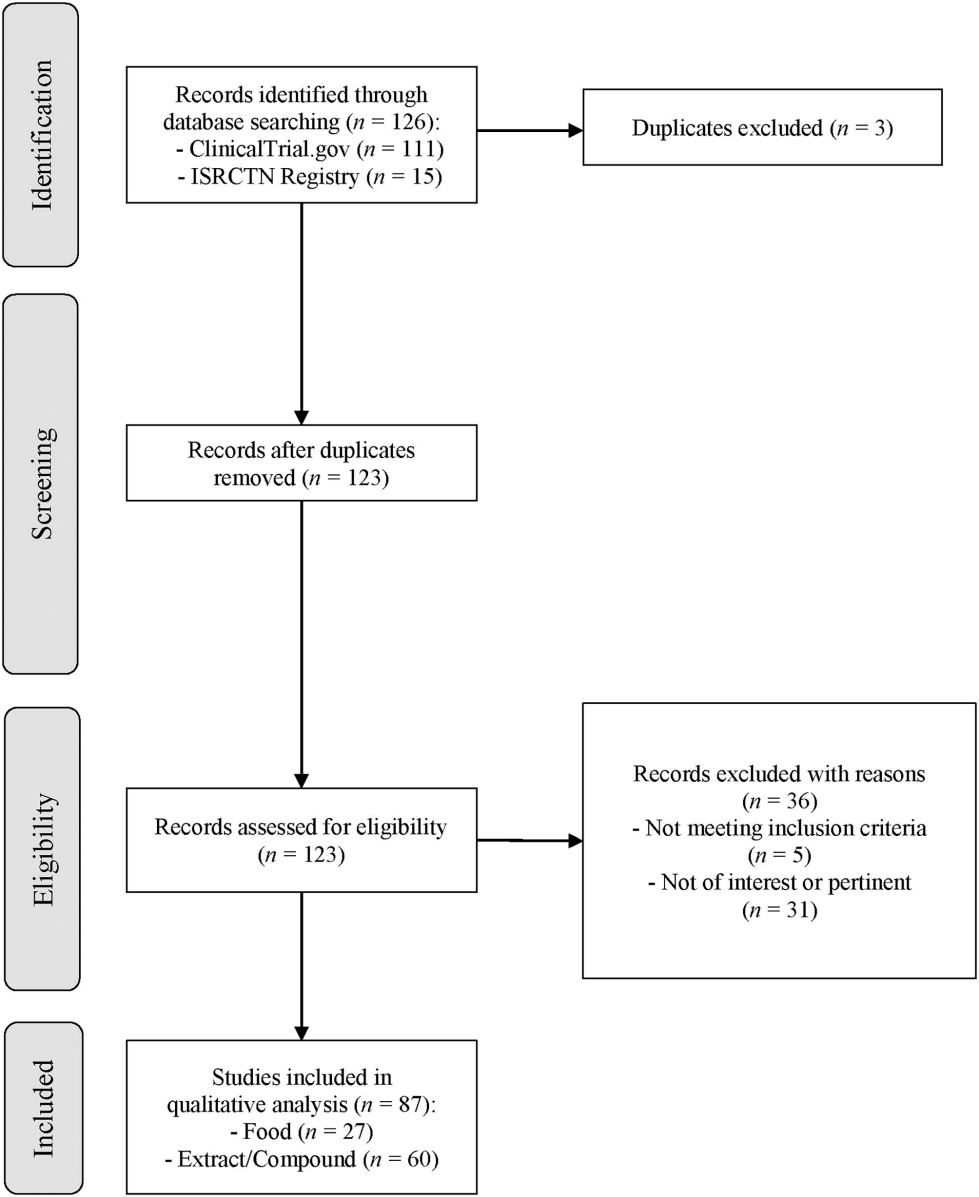
Flow diagram of the literature search process. Legend: ISRCTN, International Standard Randomized Controlled Trial Number.

The search strategy is summarized in Figure 1. Studies were considered eligible if they performed human intervention studies investigating the bioavailability of GSLs and/or their effects on any marker of human health. The only exclusion criteria adopted was the use of GSLs in combination with other nutrients or dietary bioactives or drugs in order to select specific studies focused on the effects of GSLs. No restrictions related to the country and the characteristics of the participants were applied.

A detailed list of eligibility criteria, developed by following the Population, Intervention, Comparison, Outcome, Study design (PICOS) format is provided in Table 1. Two independent reviewers (MM and CDB) conducted the study selection in the scientific databases and evaluated the eligibility of the clinical trials. Discrepancies between reviewers were solved through consultation with a third independent reviewer (DM) to achieve a consensus.

**Table 1:** Population, intervention, comparison, outcome, study design (PICOS) criteria for trial selection cited.

**Parameter**	**Criteria**
Population (P)	Healthy or diseased children, adults and/or older adults
Intervention (I)	Food, extract or pure GSLs tested alone. No other bioactive compound or drug
Comparison (C)	Control group without GSLs
Outcome (O)	Any effect on human health and bioavailability
Study design (S)	No restriction on study design

*GSLs, glucosinolates*.

### Data Extraction

Two reviewers (MM and CDB) performed data extraction. A third reviewer (DM) checked the extracted information in order to ensure the accuracy of the data reported. For each study, the following information was collected: registration number, registration year, location, funding, participant information, study design, intervention, health condition, outcome measures, and publications derived from the trials. Since no registered clinical trials were present before 2000, studies were classified into 4 main categories based on their start date (2000–2004, 2005–2009, 2010–2014, and 2015–2021). Within these different time intervals, all studies were then further divided into two sections: GSL-rich foods and GSL-rich extracts or single pure compounds. Regarding the study location, countries were classified as “low” (number of registered studies in that country <5), “medium” (5–10), and “high” (higher than 10) in accordance with previous investigations (36). Moreover, regarding the publications generated from the registered trials, the following data were extracted: name of first author, year of publication, country, registered trial number, study design, study population, duration of intervention, type of food or supplement, food characterization, primary outcome, and main findings.

## Results

### Study Selection

The flow diagram of the literature search process for each registry is reported in Figure 1. The keywords utilized for the search string led to the identification of 126 clinical trials registered in ClinicalTrials.gov and the ISRCTN registry. In detail, 111 resulted from ClinicalTrials.gov, and 15 resulted from the ISRCTN registry. After the exclusion of 3 duplicates, 123 records were evaluated for eligibility and a total of 36 clinical trials were eliminated from the review. In particular, 31 studies were not pertinent with the focus of our study since they did not investigate the bioavailability or health effect of GSLs, and 5 clinical trials did not meet inclusion criteria due to the use of GSLs in combination with other compounds. Finally, 87 records were included in the qualitative analysis in order to collect the main characteristics of the clinical trials, as shown in Table 2. Overall, most of the studies were performed by using extracts or pure compounds while the main outcomes were related to the health effects followed by the bioavailability of GSLs and derivatives. The number of studies conducted on healthy subjects was similar to those carried out on patients, while chronic interventions were more frequent than the acute ones. As reported in Table 2, most of the registered clinical trials were developed in United States (*n* = 51), both for foods and extracts, and the main primary outcomes investigated were those related to cognitive function, cancer, and urinary GSL metabolites concentration.

**Table 2:** The number of registered trials on GSL-rich foods and extracts or pure compounds, according to their characteristics.

	**Foods (*n* = 27)**	**Extracts or Pure Compounds (*n* = 60)**
**Goal**		
Health effects	18	47
Bioavailability	8	6
Both	1	7
**Duration**		
Acute	8	2
Chronic	18	55
Both	1	3
**Subjects**		
Healthy	17	24
Subjects with risk factors	3	2
Patients	7	34
**Primary outcome**		
Cardiovascular risk markers	1	1
Glucose and insulin parameters	0	2
Vascular and endothelial function	0	2
Cognitive function	0	15
Inflammation	5	2
Gut microbiota	2	0
Safety and tolerability	1	5
Detoxification	2	9
Blood GSLs concentration	4	4
Urinary GSLs concentration	7	8
Cancer	4	11
Oxidative stress	0	4
Skin health markers	0	3
Other	7	5
**Location**		
United States of America	14	37
United Kingdom	10	6
China	0	11
Sweden	0	3
Spain	2	0
Portugal	1	0
Brazil	0	1
Denmark	0	1
Japan	0	1

*GSLs, glucosinolates*.

### Trials on GSL-Rich Foods and Extracts/Pure Compounds

**Figure 2:**
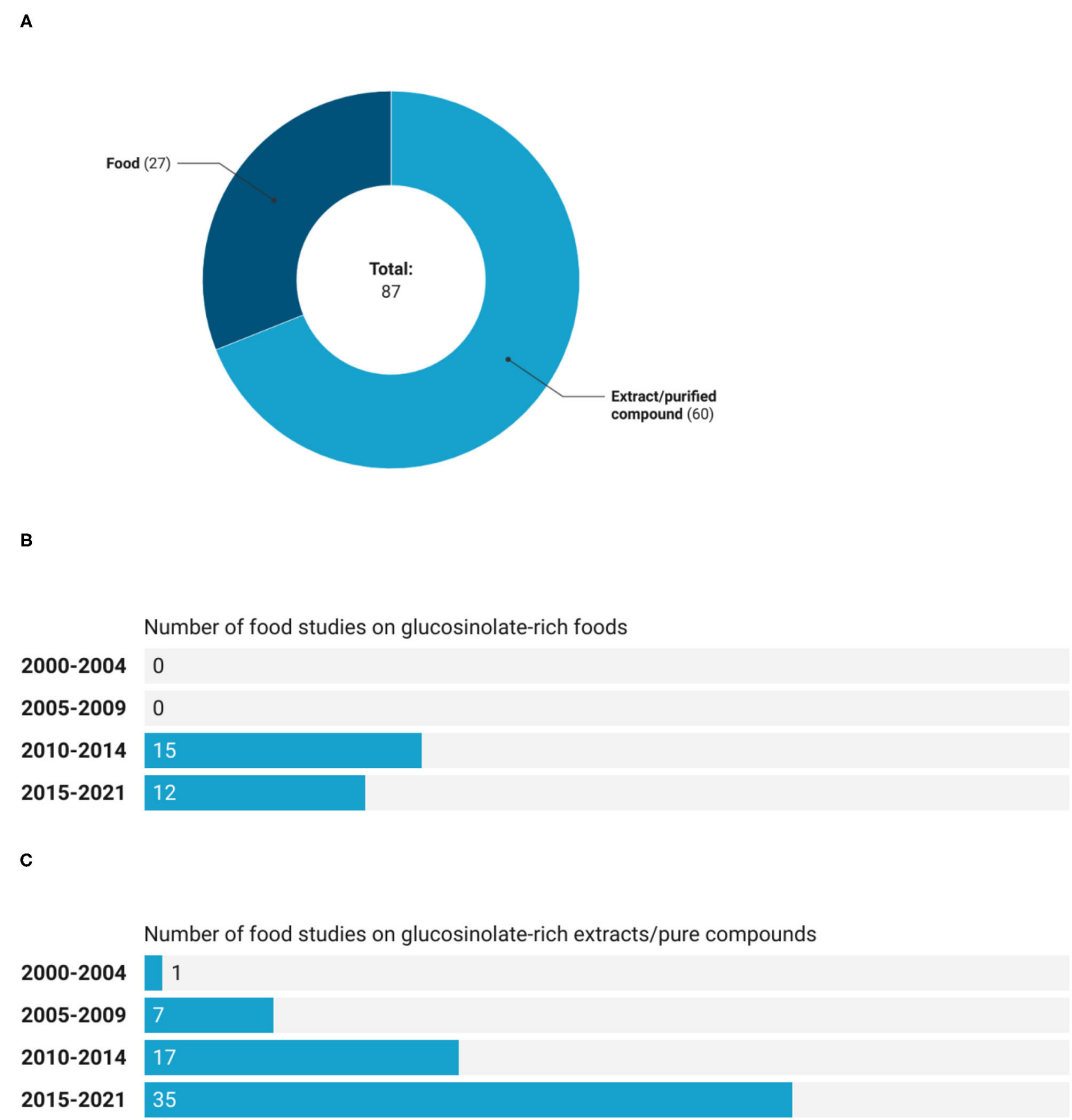
Number **(A)** and trend of registered trials on GSL-rich foods **(B)**, and extracts or pure compounds **(C)**.

Considering the whole period, from January 2000 to April 2021, registered clinical trials on GSLs that used extracts or pure compounds were 69% of the total number vs. 31% of studies that investigated the effect of GSL-rich foods (Figure 2A). Further, as shown in Figure 2B, the first clinical trial regarding a GSL-rich food was registered in the time period 2010–2014, while a study on GSL-rich extract was already reported in the time interval 2000–2004 (Figure 2C). Moreover, in the time intervals considered, the number of studies performed on extracts or pure compounds was higher compared to those executed on GSL-rich foods. Additionally, these latter studies did not show a growing trend since during the last period (from 2015 to 2021), less studies were registered compared to 2010–2014. On the contrary, more than 50% (35 records out of 60) of registered clinical trials have been using GSLs' extracts or pure compounds between 2015 and 2021.

#### Types of GSL-Rich Foods

**Figure 3:**
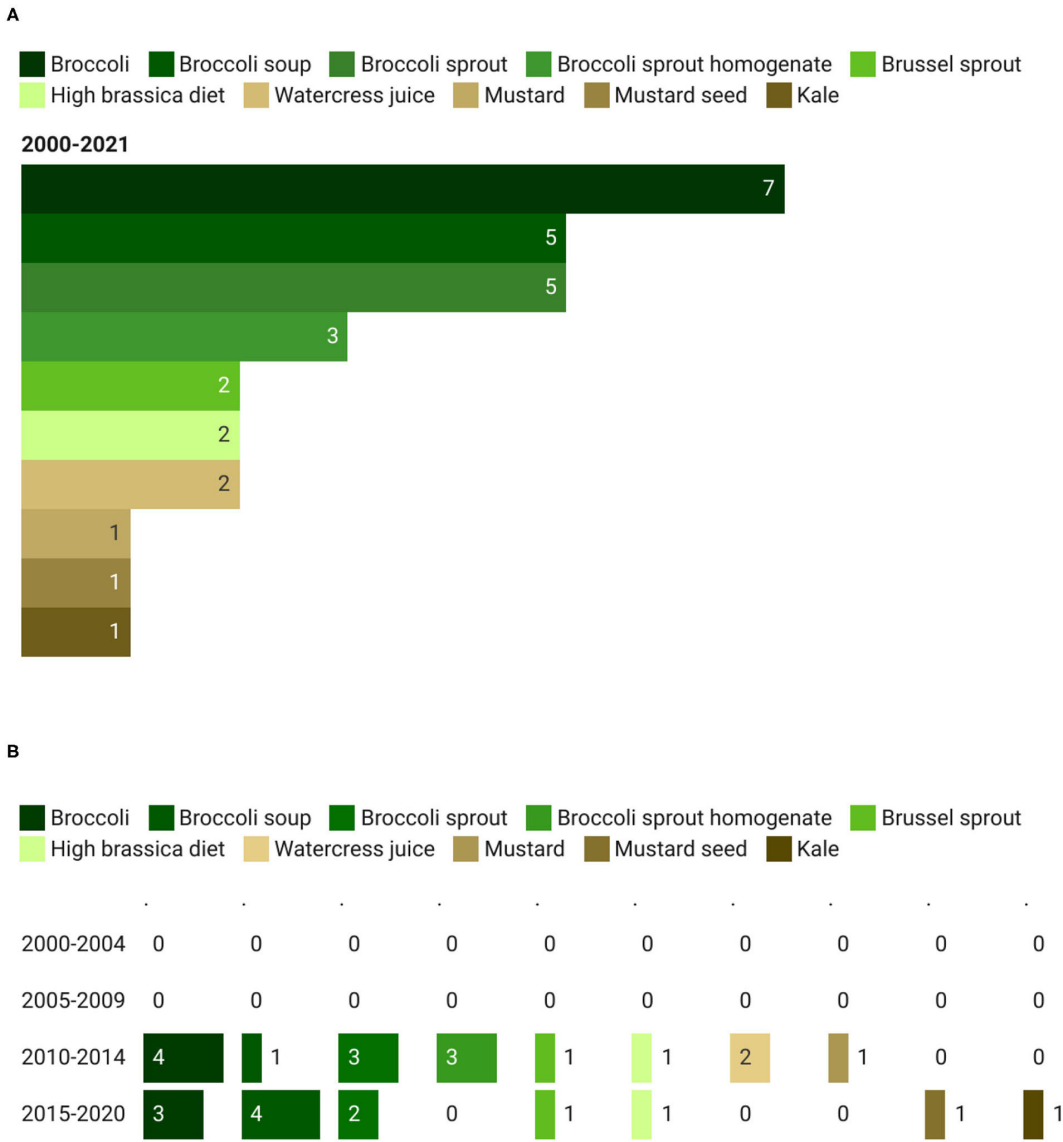
Main glucosinolates (GSL)-rich foods **(A)** used in clinical trials and their trend **(B)**. The total count of GSL-rich foods is higher than the total count of the corresponding studies since in some studies more than one food has been provided to the participants.

As reported in Figure 3A, the most studied GSL-rich food was represented by broccoli and broccoli sprouts, with 20 out of 27 clinical trials (74%) registered in ClinicalTrials.gov and ISRCTN registry. In detail, 7 records were on whole broccoli, 5 on broccoli soup, 5 on broccoli sprouts, and 3 on broccoli sprout homogenate (BSH). Other GSL-rich foods considered were mustard (14.8%), Brussel sprouts (7.4%), watercress (7.4%), and kale (7.4%). Only one study investigated the effect of a high-brassica diet. Regarding the trend, the proportion of studies on broccoli compared to other foods was similar between the time periods, 68.7% (corresponding to 11 out of 16 studies) from 2010 to 2014, and 69.2% (9 out of 13 studies) between 2015 and 2021 (Figure 3B).

#### Types of GSL-Rich Extracts

**Figure 4:**
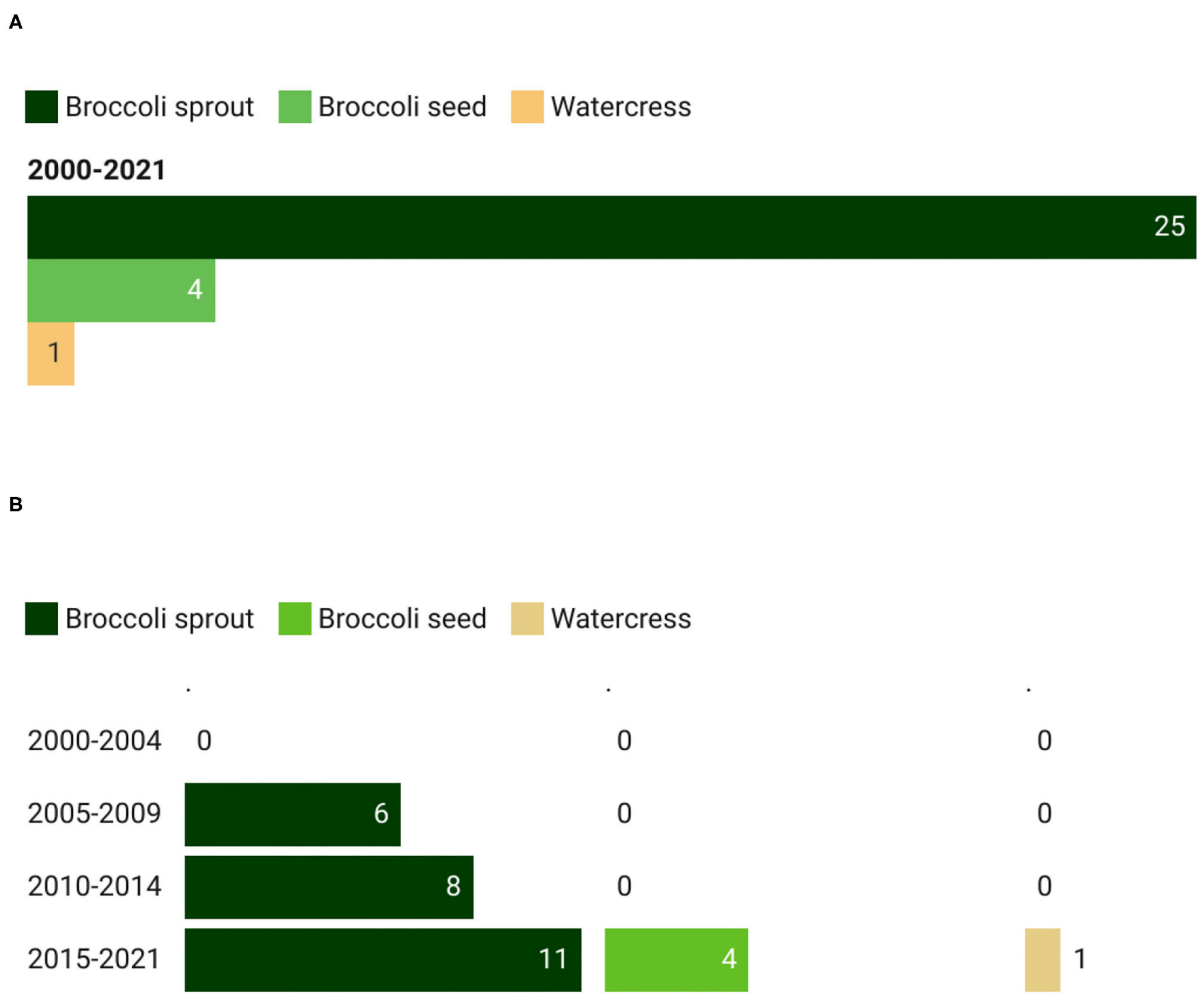
Main GSL-rich extracts **(A)** used in clinical trials and their trend **(B)**. The total count of GSL-rich extracts is higher than the total count of the corresponding studies since in some studies more than one extract has been provided to the participants.

In a similar manner to the intervention studies on GSL-rich foods, broccoli extract was the most studied, accounting for 83.3% of the registered trials (Figure 4A). Moreover, clinical trials on broccoli extracts observed a constant increase over the years, ranging from 6 registrations between 2005 and 2009 to 11 records between 2015 and 2021 (Figure 4B). During the last time interval (from 2015 to 2021), other food extracts consisting of broccoli seeds (13.3%) and watercress (3.3%) were used.

#### Types of Pure GSLs

**Figure 5:**
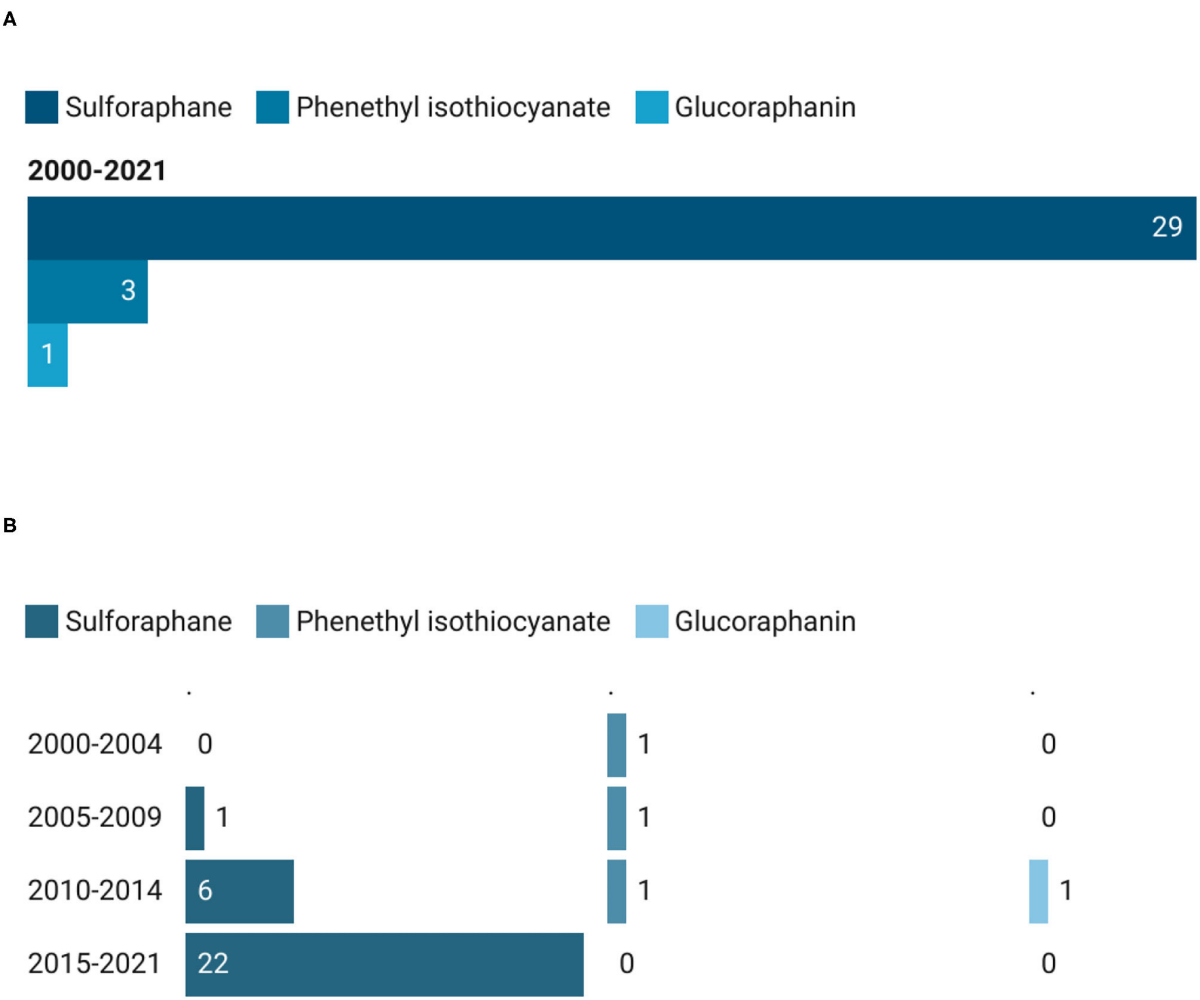
Main pure GSLs used in clinical trials **(A)** and their trend **(B)**. The total count of pure GSLs is higher than the total count of the corresponding studies since in some studies more than one GSL has been provided to the participants.

Among the 33 intervention studies that were conducted by testing pure compounds, 29 trials (87.9%) were on sulforaphane (SFN), 3 (9.1%) on phenethyl isothiocyanate (PEITC), and only 1 (3%) on glucoraphanin (GR) (Figure 5A). Most of the clinical trials which used SFN as extract were registered during the last time period (22 out of 29), showing a growing trend (Figure 5B). From 2000 up to 2004, only 1 study has been recorded, and it was on PEITC. On the other hand, the same compound did not observe an increasing interest over the years, maintaining 1 study for each time period and no records during the last time interval, from 2015 until today.

### Characteristics of Subjects

**Figure 6:**
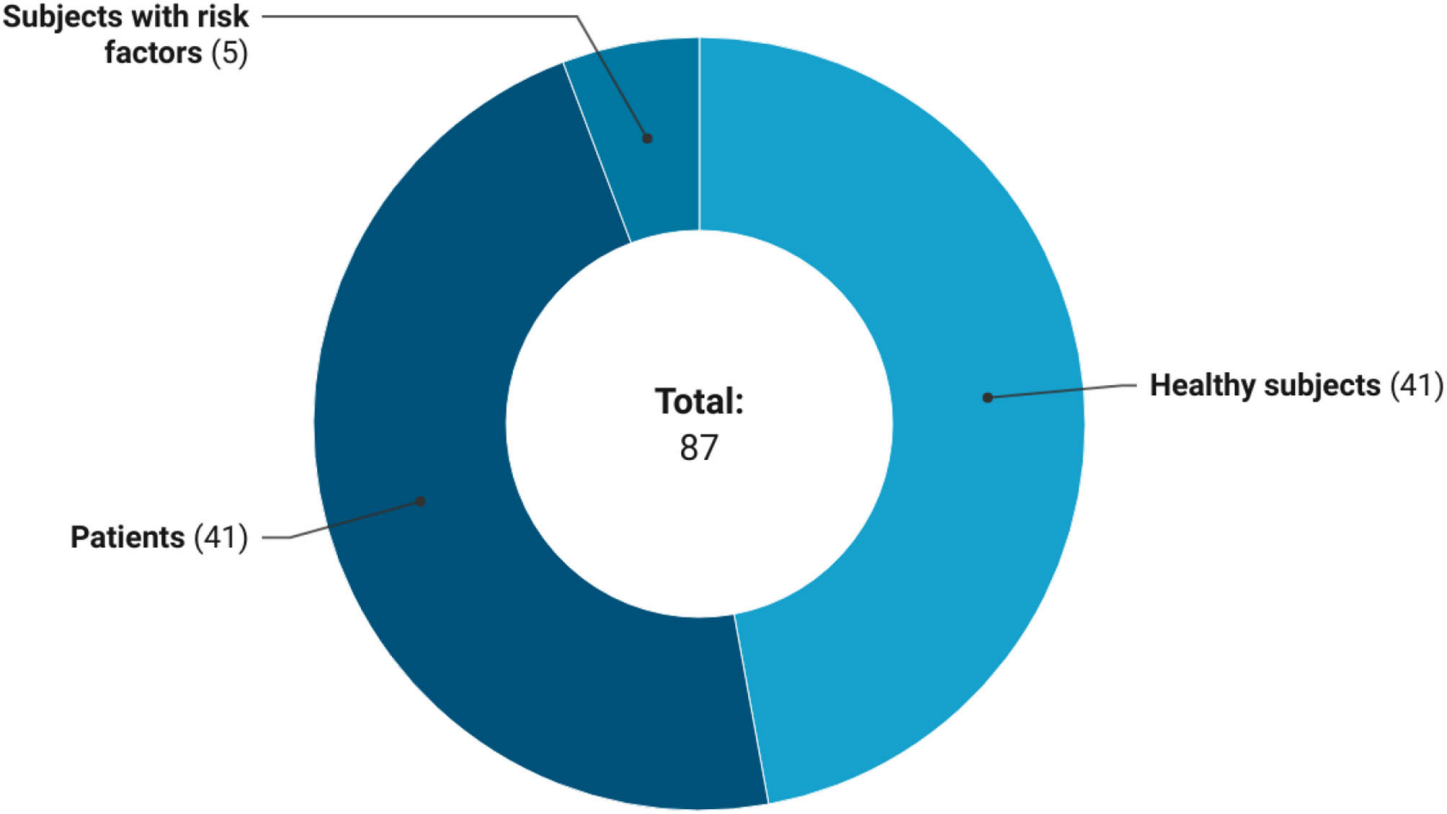
Health status of subjects included in the registered clinical trials.

In relation to the characteristics of the participants (Figure 6), trials on GSLs were conducted both on healthy subjects (*n* = 41 studies, 47.1%) and subjects with diseases such as cancer, Alzheimer's disease, diabetes mellitus, depressive disorder, and autism spectrum disorder (*n* = 41 studies, 47.1%). While, only five (5.7%) clinical trials enrolled subjects with cardiovascular risk factors, such as overweight, pre-hypertension, hypercholesterolemia, and smokers. The enrollment of participants with diseases was more frequent in clinical trials that provided extracts than studies on GSL-rich foods (34 and 7 studies, respectively). On the contrary, interventions with foods involved mainly healthy subjects than trials on GSL-rich extracts (63 and 40%, respectively). Regarding the age of participants, 46 (53.5%) registered clinical trials included both adults and older subjects, 32 (37.2%) studies included only adults, 6 (7%) studies focused on children, while 2 (2.3%) studies included only older individuals.

### Principal Purposes of the Registered Trials

**Figure 7:**
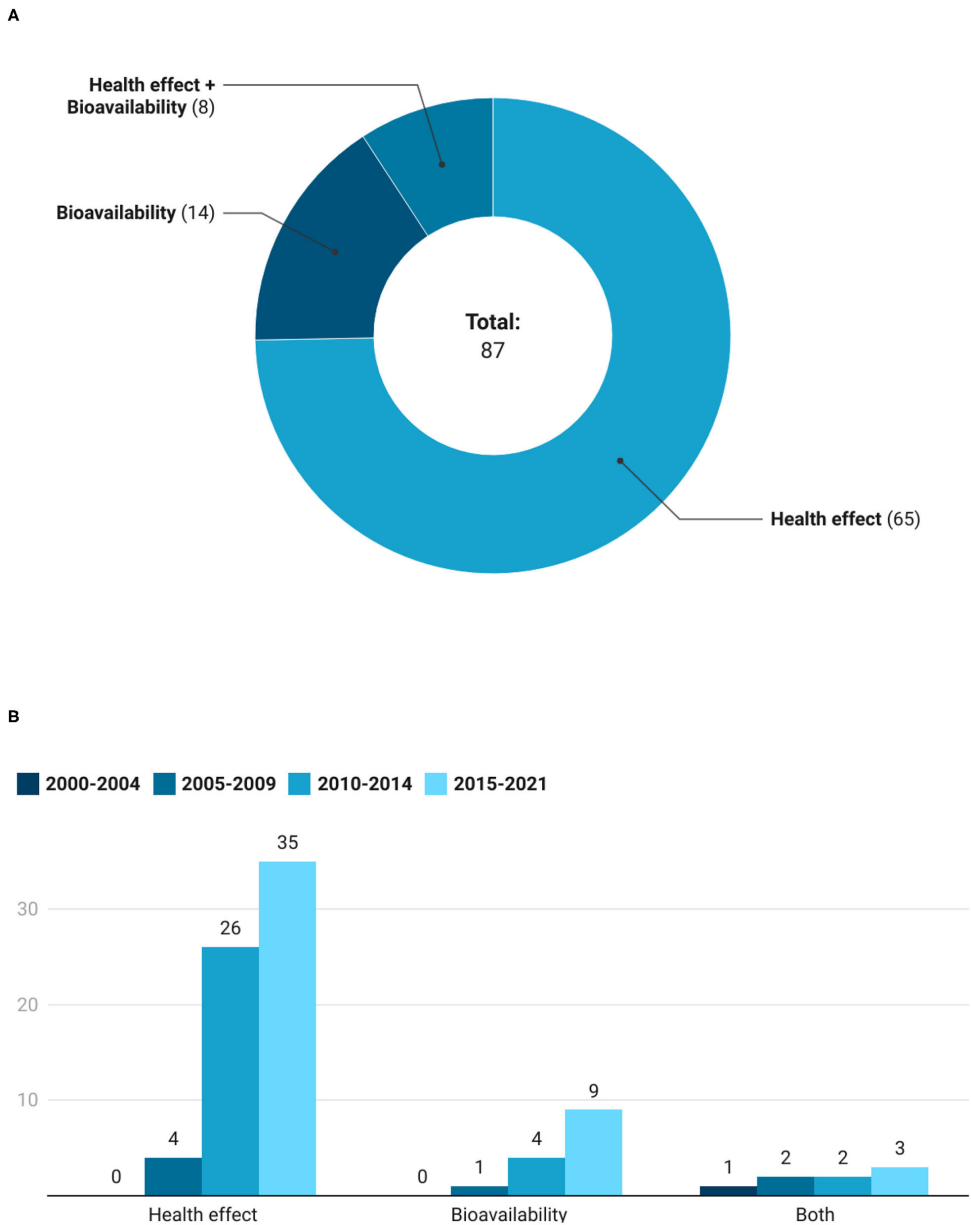
Number **(A)** and trend **(B)** of studies that assess bioavailability, health effect, and both bioavailability and health effect of GSLs.

The main objectives of the registered clinical trials are reported in Figure 7A. Most of the trials were focused on the evaluation of the health effects of extracts and foods, followed by studies on bioavailability. In particular, 65 clinical trials (18 on foods and 47 on extracts) were concerned with the health effects, 14 (8 on GSL-rich foods and 6 on extracts) were focused on bioavailability, while 8 (1 on GSL-rich foods and 7 on extracts) evaluated both. As depicted in Figure 7B, intervention studies having as an outcome the evaluation of the health effects increased over time, moving from 4 registered trials in the period 2005–2009, until 35 studies in the period 2015–2021. Similarly, studies on bioavailability increased throughout the time periods considered, starting from only 1 registered between 2005 and 2009 until 9 studied recorded from 2015 to 2021. While studies investigating both health effects and bioavailability remained limited and stable across the time intervals, with an average of 2 registrations every 5 years.

**Figure 8:**
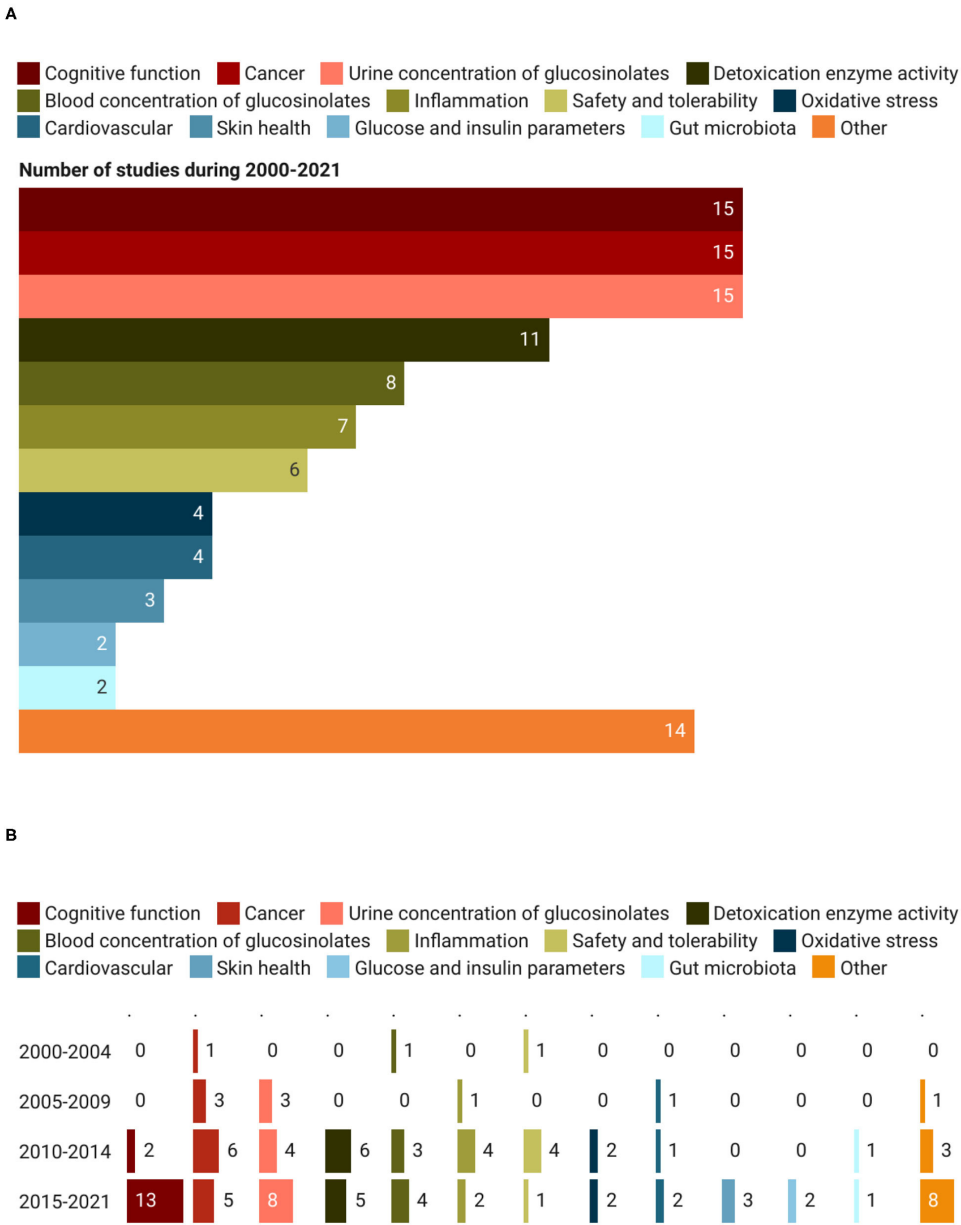
Primary outcomes **(A)** and their trend **(B)** assessed during clinical trials on GSLs.

The primary outcomes assessed in the registered trials are shown in Figure 8A. Markers of cognitive function, cancer, and bioavailability were the most analyzed. In detail, they were reported in 45 records out of 87 (15 studies for each category), accounting for 51.7% of all studies, followed by the studies focused on the activity of detoxification enzymes (11 trials; 12.6%). A similar number of studies were devoted to assessing GSL blood concentration (*n* = 8) and the role of foods and extracts on inflammation (*n* = 7) and safety/tolerability markers (*n* = 6). Other primary outcomes analyzed within the trials included: oxidative stress (*n* = 4), cardiovascular risk (*n* = 4), skin health (*n* = 3), gut microbiota (*n* = 2), and glucose/insulin parameters (*n* = 2). Among the group “Other,” reported in Figure 8A are included primary outcomes investigated only in one record out of the 87 assessed and some of them are the effect of GSLs on osteoarthritis, hormone metabolism, energy expenditure, and drug interaction (Figure 8A). This last category of outcomes “Other” observed a growing trend over the years, from 0 records in the period 2000–2004, to 1, 3, and 8 in the periods 2005–2009, 2010–2014, and 2015–2021, respectively (Figure 8B). Another category of outcomes that received greater attention only in recent years was cognitive function. During the period 2015–2021, 13 studies were registered while only 2 were found from 2010 to 2014. Similarly, the studies on bioavailability registered an increasing trend. In particular, the assessment of GSLs and their metabolites in urine and blood moved from 1 trial recorded in 2000–2004, to 3, 7, and 12 studies registered in the period 2005–2009, 2010–2014, and 2015–2021, respectively. Regarding outcomes related to the detoxification enzyme activity, cancer risk, inflammation, safety, and tolerability, the number of studies on these topics registered a decrease in the last period (2015–2021) compared to the previous one (2010–2014), moving from a total of 20 records to 11 (Figure 8B).

### Other Characteristics of the Registered Trials

**Figure 9:**
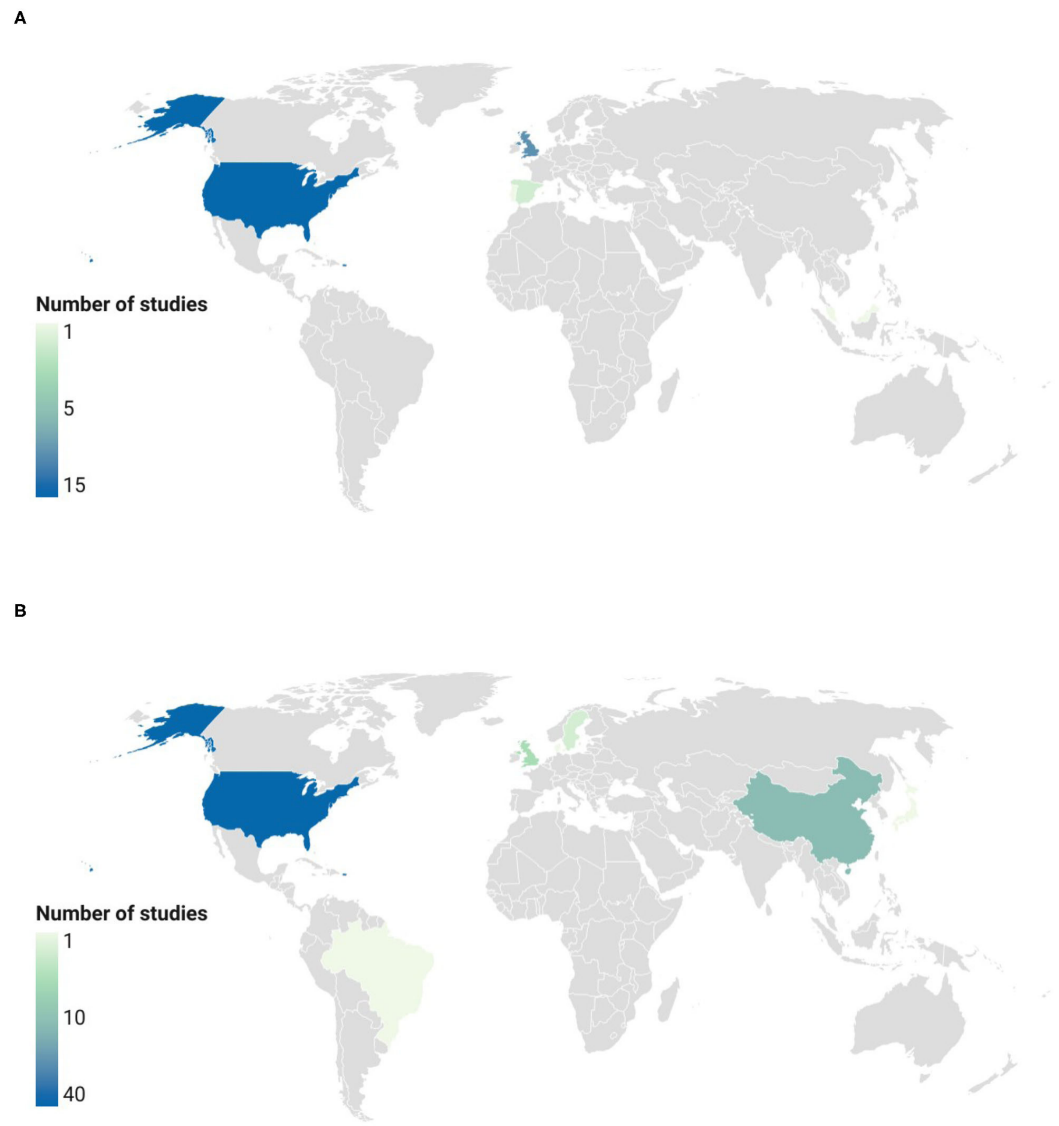
Countries with the highest number of registered studies on GSL-rich foods **(A)** and extracts **(B)**. “Low”: <5 registered trials; “medium”: 5–10 registered trials; “high”: >10 registered trials. Locations not reporting clinical trials on GSLs are colored in gray.

The main countries in which clinical trials were conducted are depicted in Figure 9. The highest number of registered studies on foods and extracts was observed in the USA (51 clinical trials: 37 on extracts and 14 on GSL-rich foods). UK and China were the countries with the highest number of studies on GSL-rich foods (16 trials: 6 on extracts and 10 on foods) and extracts (11 trials: 11 on extracts and 0 on foods), respectively. Other countries included: Sweden (*n* = 3), Spain (*n* = 2), Brazil (*n* = 1), Denmark (*n* = 1), Japan (*n* = 1), and Portugal (*n* = 1).

Regarding the type of funding, 65 studies (74.7% of the total number) received government funding, 48 were on extracts or pure compounds, and 17 were on GSL-rich foods. Private funding was allocated to 20 clinical trials, 9 on foods and 11 on extracts, while only 2 studies were funded with both government and public funding.

### Characteristics and Results of the Publications Derived From Registered Trials

Among the 87 registered trials, 28 publications were directly associated with ClinicalTrials.gov and ISRCTN registry. The main characteristics and results of the 28 publications are reported in Tables 3, 4. Out of these 28 studies, 16 were performed on GSL-rich foods (Table 3) while 12 were on GSL-rich extracts and/or pure compounds (Table 4).

**Table 3:** Characteristics and findings of publications associated with registered trials on GSL-rich foods.

**Reference, country, registry ID**	**Study design**	**Study population**	**Duration of intervention**	**Food or supplement intervention**	**Control or placebo intervention**	**Primary outcome and other variable outcomes**	**Main findings**
Christiansen et al. (37), Denmark, NCT00252018	Randomized, parallel, controlled, single-blind	*n* = 41 hypertensive subjects, non-smoker and without diabetes or hypercholesterolemia Control group: *n* = 20 (15F, 5M) age 54 ± 10 y, BMI 26.2 ± 3.2 kg/m^2^ Intervention group: *n* = 21 (10F, 11M) age 58 ± 9 y, BMI 29.1 ± 6.6 kg/m^2^	4 weeks	10 g dried broccoli sprouts daily (25.9 ± 8.5 μmol/g dry-weight GR; 48.5 ± 14.2 μmol/g dry-weight total GSL)	Habitual diet	FMD (primary outcome), BP, HDL-C, LDL-C	↔
Armah et al. (38), UK, NCT01114399	Randomized, 3-arm parallel, controlled	*n* = 48 CVD risk subjects Control group: *n* = 10 (5M) age 62.0 ± 2.12 y, BMI 25.4 ± 2.79 kg/m^2^; (5F) age 61.4 ± 2.51 y, BMI 26.4 ± 3.73 kg/m^2^ HG broccoli group: *n* = 19 (10M) age 59.8 ± 7.28 y, BMI 25.8 ± 2.99 kg/m^2^, 1 smoker; (9F) age 63.8 ± 7.92 y, BMI 25.1 ± 3.73 kg/m^2^ Standard broccoli group: *n* = 19 (9M) age 57.3 ± 5.83 y, BMI 24.6 ± 3.17 kg/m^2^, 1 smoker; (10F) age 60.8 ± 5.31 y, BMI 26.0 ± 3.20 kg/m^2^, 1 smoker	12 weeks	High-GR broccoli group: 400 g High-GR broccoli every week (21.6 ± 1.60 μmol/g dry-weight GR and 4.5 ± 0.34 μmol/g dry-weight glucoiberin) Standard broccoli group: 400 g of a standard broccoli cultivar every week (6.9 ± 0.44 μmol/g dry-weight GR and 0.7 ± 0.33 μmol/g dry-weight glucoiberin)	400 g garden peas every week	TC (primary outcome), SBP, DBP, HDL-C, LDL-C, TG, ox-LDL, hsCRP, PWV, AIx	↔
Noah et al. (39), USA, NCT01269723	Randomized, parallel, placebo-controlled, double-blind	Healthy subjects Smoker ASH group: *n* = 10 (3F, 7M) age 28.1 ± 1.3 y, BMI 27.6 ± 2 kg/m^2^ Smoker BSH group: *n* = 6 (2F, 4M) age 27.3 ± 1.7 y, BMI 27.2 ± 1.4 kg/m^2^ Non-smoker ASH group: *n* = 20 (14F, 6M) age 26.9 ± 1.3 y, BMI 24.9 ± 0.8 kg/m^2^ Non-smoker BSH group: *n* = 15 (7F, 8M) age 26 ± 1.3 y, BMI 25.5 ± 0.9 kg/m^2^	4 days	200 g BSH Composition: NA	200 g ASH	IL-6 (primary outcome), influenza B RNA sequence quantity in NLF cells NQO1 mRNA in NLF cells IL-8, IP-10, INF-γ, HO-1 mRNA in NLF cells Influenza B RNA sequence quantity, HO-1 and NQO1 mRNA in nasal biopsy	↓ in smokers ↑ in smokers ↔
Doss et al. (40), USA, NCT01715480	Non controlled, baseline and post-intervention	*n* = 14 patients with sickle cell disease 50 g BSH group: *n* = 5 (2F, 3M) age 31.8 ± 5.5 y 100 g BSH group: *n* = 5 (2F, 3M) age 37.4 ± 9.3 y 150 g BSH group: *n* = 4 (3F, 1M) age 35.5 ± 11.2 y	21 days	50, 100, or 150 g BSH daily Composition: NA	NA	Vital signs (primary outcome), adverse signs (primary outcome), HbF (primary outcome) Nrf2 genes expression in sickle cells Measurement of blood chemistries, cell counts, LDH (primary outcome)	↔ ↑ HO-1 with 150g and HBG1 with 100g ↓ white blood cell with 150g
Duran et al. (41), USA, NCT01625130	Randomized, crossover, placebo-controlled, triple-blind	*n* = 16 healthy non-smoker subjects, age 18–50 y	3 days	200 g BSH daily Composition: NA	200 g ASH daily	% neutrophils in induced sputum (primary outcome), gene expression HO-1, NQO-1, GSTM-1 Plasma levels of SFN, SFN-NAC, SFN-GSH	↔ ↑
Muller et al. (42), USA, NCT01269723	Randomized, parallel, placebo-controlled-double-blind	*n* = 29 non-smokers healthy subjects ASH group: *n* = 16 (12F, 4M) age 27.6 ± 1.5 y, BMI 25.1 ± 1.0 kg/m^2^ BSH group: *n* = 13 (7F, 6M) age 25.5 ± 1.5 y, BMI 25.5 ± 1.1 kg/m^2^	4 days	200 g BSH (100 μmol SFN)	200 g ASH	NKT cells in peripheral blood T cell, NK cells, monocytes, macrophages, neutrophils in the peripheral blood, markers of systemic NK cells Granzyme B production in NK cells	↓ ↔ ↑
Sudini et al. (43), USA, NCT01183923	Randomized, crossover, placebo-controlled, triple-blind	*n* = 40 asthmatic adults, non-smoker Broccoli sprouts group: *n* = 20 (11F, 9M) age 3.1 ± 9.62 y, BMI 30.9 (25.1–37.0) kg/m^2^ Placebo group: *n* = 20 (13F, 7M) age 34.2 ± 9.17 y, BMI 32.2 kg/m^2^	3 days	100 g broccoli sprouts daily	100 g alfalfa sprouts daily	Change in FENO (primary outcome), FEV1, FVC, asthmal control test score, rhinitis quality of life score, levels of HO-1, NQO1, GCLC and GCLM, antioxidant gene expression in PBMCs, urinary isoprostane, serum TBARS levels, protein carbonyl levels, IL-4, IL-13, IL-6 levels	↔
Langeveld et al. (44), UK, ISRCTN19147515	Randomized, crossover, placebo-controlled, single-blind	*n* = 11 healthy non-smoker subjects (5F) age 33.7 ± 6.9 y, BMI 22.9 ± 0.9 kg/m^2^; (6M) age 41.6 ± 12.2 y, BMI 22.5 ± 1.5 kg/m^2^	3 separate days	10 g capsulated mustard 10 g mustard (~1.6 mg allyl-ITC/g mustard)	10 g capsulated placebo mixture Composition: tomato ketchup, olive oil, water	EE (primary outcome), substrate oxidation, core temperature, cold scores hunger scores, EI, plasma glucose, plasma non-esterified fatty acids, plasma free thyroxin, plasma cortisol	↔
Nguyen et al. (45), UK, NCT01357070	Randomized, crossover, controlled, double-blind	*n* = 6 healthy non-smokers subjects (4F, 2M) age 26.5 (24.5–29.3) y, BMI 20.2 (19.1–21.3) kg/m^2^	24 h	200 g of BSH	200 g of ASH	ROS activation in leukocytes (primary outcome), p38 MAP kinase phosphorylation in leukocytes (primary outcome) p65 Nf-kB activation in leukocytes (primary outcome)	↓ ↔
Charron et al. (46), USA, NCT02346812	Randomized, crossover, controlled, single-blind	*n* = 18 healthy subjects	18 days	100 g broccoli and 10 g daikon radish 2 times daily for 15 days On day 16 100 g broccoli and 10 g daikon radish 1 time On day 17 200 g broccoli, 20 g daikon radish, 100-g roll and 10 g margarine (97.5 μmol GR and 5.8 μmol glucoerucin in 200 g of broccoli)	Diet without broccoli or other Brassica vegetables	Plasma total ITCs metabolites AUC (primary outcome), Cmax Plasma total ITCs metabolites k Urine total ITCs metabolites (primary outcome), plasma total ITCs metabolites Tmax, lactulose:mannitol ratio	↑ subjects with BMI <26 kg/m^2^ ↓ ↔
Lòpez-Chillòn et. al. (47), Spain, NCT03390855	Non controlled, baseline and post-intervention	*n* = 40 overweight subjects (21M, 19F) age 46 ± 6 y, BMI 28.9 ± 4.0 kg/m^2^	10 weeks	30 g fresh broccoli sprouts daily (51 mg GR, 20 mg neoglucobrassicin and 121.11 mg total GSL per dose)	NA	Plasma levels of IL-6 and CRP (primary outcome), FM Body weight, BMI Urine GSL and ITCs level	↓ ↔ ↑
Sivapalan et al. (17), UK, NCT02300324	Randomized, crossover, placebo-controlled, double-blind	*n* = 10 healthy subjects (7F, 3M) age 42.9 ± 17.4 y, BMI 25.2 ± 3.2 kg/m^2^	3 separate days	Group 2: 300 g Myb28^B/V^ broccoli (Beneforte broccoli) and stilton soup (280 ± 8.8 μmol GR per portion) Group 3: 300 g Myb28^V/V^ broccoli and stilton soup (452 ± 10.6 μmol GR per portion)	Group 1: 300 g Myb28^B/B^ broccoli (standard broccoli) and stilton soup (84 ± 2.8 μmol GR per portion)	Total excretion of SFN and metabolites in 24 h urine (primary outcome), plasma level of SFN and its metabolites AUC, Cmax, Tmax, total excretion of GR and glucoerucin in urine, AUC, Cmax	↑
Chen et al. (48), China, NCT02656420	Randomized, parallel, placebo-controlled, single-blind	*n* = 170 healthy subjects Placebo group: *n* = 55 (38F, 17M) age 56 (53–61) y, BMI 25.1 (23.1–26.7) kg/m^2^ 1/5 dose group: *n* = 55 (43F, 12M) age 60 (53–63) y, BMI 24.7 (22.6–26.8) kg/m^2^ 1/2 dose group: *n* = 35 (28F, 7M) age 58 (50–64) y, BMI 23.9 (22.1–26.6) kg/m^2^ Full dose group: *n* = 25 (18F, 7M) age 59 (56–62) y, BMI 23.5 (21.6–25.8) kg/m^2^	10 days	Full dose: 100 ml broccoli sprouts beverage with pineapple juice, lime juice and water, nightly (600 μmol GR and 40 μmol SFN) 1/2 dose: 100 ml broccoli sprouts beverage with pineapple juice, lime juice and water, nightly (300 μmol GR and 20 μmol SFN) 1/5 dose: broccoli sprouts beverage with pineapple juice, lime juice and water, nightly (120 μmol GR and 8 μmol SFN)	100 ml beverage containing pineapple juice, lime juice and water. Nightly	Urinary excretion of S-phenylmercapturic acid	↑ full dose
Coode-Bate et al. (49), UK, NCT02821728	Randomized, parallel, controlled	*n* = 18 healthy males scheduled for prostate biopsy Control group: *n* = 9, age 64.7 ± 5.39 y, BMI 26.8 ± 3.29 kg/m^2^ Supplementation group: *n* = 9, age 68.8 ± 6.46 y, BMI 28.1 ± 2.58 kg/m^2^	4 weeks	300 g GR-rich broccoli soup per week (280 ± 8.8 μmol GR per portion and 1,513 ± 36.8 μmol of SMCSO)	Habitual diet	Sulfate levels in prostate tissues (primary outcome), SMCSO in periprostatic adipose tissue SMCSO levels in prostate tissues, SMCSO in urine	↔ ↑
Traka et al. (11), UK, ISRCTN40496794	Randomized, 3-arm parallel, placebo-controlled, double-blind	*n* = 49 males with diagnosis of low-risk or intermediate-risk prostate cancer Soup X group (control): *n* = 16, age 68 ± 5 y, BMI 26.7 ± 3.1 kg/m^2^ Soup Y group: n = 17, age 66 ± 6 y, BMI 27.6 ± 3.4 kg/m^2^ Soup Z group: n = 16, age 66 ± 6 y, BMI 27.7 ± 2.2 kg/m^2^	12 months + optional 12 months	Broccoli soup Y (genotype Myb28 B/V): 300 ml weekly (214 ± 7.3 μmol GR) Broccoli soup Z (genotype Myb28 V/V): 300 ml weekly (492 ± 3.2 μmol GR)	Broccoli soup X (genotype Myb28 B/B): 300 ml weekly (72 ± 2.8 μmol GR)	Prostate cancer biomarkers measured in prostate tissue (primary outcome) Metabolomics analysis	↓ ↔
Charron et al. (46), USA, NCT03013465	Randomized, crossover, controlled, single-blind	*n* = 17 healthy subjects (9F, 8M) age 54.3 ± 9.2 y, BMI 26.9 ± 4.3 kg/m^2^	16 days	200 g cooked broccoli daily, 100 g of broccoli on day 16, 200 g of broccoli, 100 g roll and 10 g of margarine on day 17 (147.6 μmol GR and 3.6 μmol glucoerucin in 200 g of broccoli)	No broccoli consumption	Percentage of total plasma AUC, plasma total metabolites Cmax, T max, k, total urinary accumulation (primary outcomes)	↔

*AIx, augmentation index; ASH, alfalfa sprouts homogenate; AUC, area under curve; BMI, body mass index; BP, blood pressure; BSH, broccoli sprouts homogenate; Cmax, maximum concentration; CRP, C-reactive protein; CVD, cardiovascular disease; EE, energy expenditure; EI, energy intake; FENO, fractional exhaled nitric oxide; FEV1, forced expiratory volume; FM, body fat mass; FMD, flow mediated vasodilatation; FVC, forced vital capacity; DBP, Diastolic blood pressure; GCLC, glutamate cysteine ligase catalytic subunit; GCLM, glutamate cysteine ligase modifier subunit; GR, glucoraphanin; GSL, glucosinolates; GSTM1, glutathione S-transferase Mu 1; HbF, fetal hemoglobin; HBG1, hemoglobin subunit gamma 1; HDL-C, high-density lipoprotein cholesterol; HO-1, heme oxygenase-1; hsCRP, high-sensitive C-reactive protein; IL-13, Interleukin 13; IL-4, Interleukin 4; IL-6, Interleukin-6; IL-8, Interleukin-8; INF-γ, Interferon gamma; IP-10, Interferon gamma-induced protein 10; ITCs, isothiocyanates; k, elimination rate constant; LDH, lactate dehydrogenase; LDL-C, low-density lipoprotein cholesterol; NA, not available; Nf-kB, nuclear factor kappa-light-chain-enhancer of activated B cells; NK, natural killer; NKT, natural killer T; NLF, nasal lavage fluid; NQ01, NADPH quinone oxidoreductase-1; Nrf2, nuclear factor erythroid-2 related factor 2; Ox-LDL, oxidized low-density lipoprotein cholesterol; PBMC, peripheral blood mononuclear cell; PWV, pulse-wave velocity; ROS, reactive oxygen species; SBP, systolic blood pressure; SFN, sulforaphane; SFN-GSH, sulforaphane-glutathione; SFN-NAC, sulforaphane-N-acetyl-L-cysteine; SMCSO, S-methyl cysteine sulfoxide; TBARS, thiobarbituric acid reactive substances; TC, total cholesterol; TG, tryglicerides; Tmax, time of maximum concentration; ↓, significant decrease; ↑, significant increase; ↔, no significant effect*.

**Table 4:** Characteristics and findings of publications associated with the registered trials on extracts or pure compounds.

**Reference, country, registry ID**	**Study design**	**Study population**	**Duration of intervention**	**Food or supplement intervention**	**Control or placebo intervention**	**Primary outcome and other variable outcomes**	**Main findings**
Poulton et al. (50), USA, NCT00621309	Randomized, crossover, controlled	*n* = 23 healthy non-smoker subjects (11F, 12M) age 23.7 y (20–37 y), BMI 23.4 ± 2.7 kg/m^2^	7 days	Group 1: 450 μmol SFN (BSE) + cheese soup dailyGroup 2: 450 μmol SFN (BSE) + cheese soup and 300 mg rifampicin daily	Group 3: cheese soup and 300 mg rifampicin daily	Pharmacokinetic Measure of Cytochrome P450 3A4 Activity (primary outcome)	↔
Haber et al. (51), USA, NCT00882115	Non controlled, baseline and post-intervention	*n* = 28 healthy subjects (14F, 14M)	4 days	1.25 g BSE (100 μmol SFN)	NA	Total number of nasal leukocytes (primary outcome)	↓
Singh et al. (52), USA, NCT01474993	Randomized, parallel, placebo-controlled, quadruple-blind	*n* = 40 males patients with ASD, age 13–27 y SFN treated group: *n* = 26 Placebo group: *n* = 14	18 weeks	SFN-rich BSE: 50 μmol SFN for weight <100 lb 100 μmol SFN for weight = 101–199 lb 150 μmol SFN for weight > 200 lb	Microcrystalline cellulose	SRS (primary outcome), ABCS CGI-I	↓ ↑
Atwell et al. (53), USA, NCT00843167	Randomized, parallel, placebo-controlled, triple-blind	*n* = 54 females with diagnostic mammogram, age 54 ± 12 y, BMI 27.3 ± 5.6 kg/m^2^	2–8 weeks	~250 mg broccoli seed extract, 2 pills 3 ×/day (224 mg GR total daily dose)	Microcrystalline cellulose 3 × /day	Urinary levels of ITCs (primary outcome) HDA Activity (primary outcome) Ki-67 (primary outcome), HDAC3 H3K9ac p21, HDAC6, H3K18ac	↑ ↓ ↓ in benign tissues in the SFN group ↓ in ductal carcinoma tissue in the placebo group ↔
Rajendran et al. (54), USA, NCT01543074	Randomized, parallel, placebo-controlled, double-blind	*n* = 10 healthy subjects BSE group: *n* = 5 Placebo group: *n* = 5	7 days	200 μmol SFN equivalent of BSE	Placebo	Blood levels of SFN and its metabolites	↑
Shiina et al. (55), Japan, NCT01716858	Non controlled, baseline and post-intervention	*n* = 10 patients with schizophrenia (6F, 4M) age 42.7 ± 11 y	8 weeks	30 mg SFN-GSL daily	NA	PNSS (primary outcome), Levels of brain-derived neurotrophic factors CogState	↔ ↑ only in the Accuracy component of the One Card Learning Task
Yuan et al. (56), USA, NCT00691132	Randomized, crossover, placebo-controlled, double-blind	*n* = 82 healthy current smokers (38F, 44M) age 41 ± 10.1 y, BMI 28 ± 5.6 kg/m^2^	5 weeks	10 mg of PEITC in 1 ml olive oil, 4 times/day, once every 4 h for 5 days	Olive oil, 4 times/day, once every 4 h for 5 days	Urinary levels of total ITCs and PEITC-NAC (primary outcome) The ratio of urinary [pyridine-D4]hydroxy acid: total [pyridine-D4]NNAL (primary outcome), urinary levels of PEITC-NAC and total ITCs by GSTM1 or both genotypes GSTM1 and GSTT1 Urinary levels of PEITC-NAC and total ITCs by GSTT1 genotypes	↑ ↓ ↔
Wise et al. (57), USA, NCT01335971	Randomized, 3-arm parallel, placebo-controlled, quadruple-blind	*n* = 89 smoker with physician diagnosed COPD subjects Placebo group: *n* = 31 (15F, 16M) age 59 (52–67) Group 1: n = 29 (12F, 17M) age 59 (54–65) y Group 2: *n* = 29 (8F, 21M) age 56 (52–62) y	1 month	Group 1: 25 μmol SFN daily Group 2: 150 μmol SFN daily	Microcrystalline cellulose	Alveolar macrophage expression of Nrf2, NQ01, HO1, AKR1C1, AKR1C3 (primary outcome), bronchial epithelial cell expression of Nrf2, NQ01, HO1, AKR1C1, AKR1C3 (primary outcome), isoprostane, TBARS in plasma and expired breath condensate, cytokine profiles in bronchoalveolar lavage fluid	↔
Axelsson et al. (58), Sweden, NCT02801448	Randomized, parallel, placebo-controlled, quadruple-blind	*n* = 97 patients with type 2 diabetes (24F, 73M) age 35–75 y, BMI 30.4 ± 4.0 kg/m^2^	12 weeks	150 μmol SFN in BSE daily	Maltodextrin sprayed with copper chlorophyllin	HbA1c (primary outcome) Fasting blood glucose BMI, Fatty liver index, liver parameters, TC, TG, Hb	↓ ↓ in obese dysregulated T2D patients ↔
Bent et al. (59), USA, NCT02654743	Non controlled, baseline and post-intervention	*n* = 15 child with ASD (3F, 12M) age 14.7 y	12 weeks	1 μmol SFN/2.2 kg body weight daily with tablets, each one containing 125 mg BSE, 50 mg dried broccoli sprouts, 15 mg ascorbic acid and microcrystalline cellulose	NA	ABCS SRS	↑ ↑
Liu et al. (60), USA, NCT02561481	Non controlled, baseline and post-intervention	*n* = 10 young males with ASD, age 9.9 y	14 days	12.5–15 mg GR (2.2 μmol SFN/kg body weight) and active plant-derived myrosinase daily	NA	NQO1, AKR1C1, HO-1, HSP70, HSP27 IL-6, IL-1β, TNF-α, Cox-2	↔ ↓
Zhang et al. (61), USA, NCT01265953	Randomized, parallel, placebo-controlled, triple-blind	*n* = 98 males at risk for prostate cancerPlacebo group: *n* = 48, age 64.9 ± 5 y, BMI 31.1 kg/m^2^BSE group: *n* = 50, age 65.7 y, BMI 28.9 kg/m^2^	4–8 weeks	2 of BSE capsules daily (200 μmol SFN)	Gelatin capsule with microcrystalline cellulose	Total urine SFN metabolites (primary outcome), Total plasma SFN metabolites (primary outcome) PBMC HDAC activity levels Tissue levels of H3K18ac, HDAC3, HDAC6 (primary outcome), Ki-67(primary outcome), p21 ARLNC1 and AMACR gene expression	↑ ↑ in subjects with prostate cancer ↔ ↓in prostate cancer tissue

*ABCS, Aberrant Behavior Checklist scale; AKR1C1, aldo-keto reductase Family 1 Member C1; AKR1C3, aldo-keto reductase Family 1 Member C3; AMACR, alpha-methylacyl-CoA racemase; ARLNC1, androgen receptor-regulated long noncoding RNA; ASD, Autism Spectrum Disorder; BMI, body mass index; BSE, broccoli sprouts extract; CGI-I, Clinical Global Impression Improvement Scale; COPD, chronic obstructive pulmonary disease; Cox2, cyclooxygenase-2; GR, glucoraphanin; GSTM1, glutathione S-transferase Mu 1; GSTT1, glutathione S-transferase theta-1; H3K18ac, acetylated histone H3 lysine 18; H3K9ac, acetylated histone H3 lysine 9; Hb, hemoglobin; HbA1c, glycated hemoglobin; HDAC, histone deacetylase; HDAC3, histone deacetylase 3; HDAC6, histone deacetylase 6; HO-1, heme oxygenase-1; HSP27, heat shock protein 70; HSP70, heat shock protein 70; IL-1β, Interleukin-1β; IL-6, Interleukin-6; ITCs, isothiocyanates; NA, not available; NQ01, NADPH quinone oxidoreductase-1; Nrf2, nuclear factor erythroid-2 related factor 2; PBMC, peripheral blood mononuclear cell; PEITC, 2-phenethyl isothiocyanate; PEITC-NAC, N-acetyl-S-(N-2-phenethylthiocarbamoyl)-L-cysteine; PNSS, Positive and Negative Syndrome Scale; SFN, sulforaphane; SRS, Social Responsiveness Scale; T2D, type 2 diabetes; TBARS, thiobarbituric acid reactive substances; TC, total cholesterol; TG, tryglicerides; TNF-α, Tumor Necrosis Factor-α; ↓, significant decrease; ↑, significant increase; ↔, no significant effect*.

#### Characteristics and Results of the Studies Performed on GSL-Rich Foods

The main characteristics of the studies performed on GSL-rich foods are reported in Table 3. Most of the studies focused on the bioavailability of GSLs and derivatives followed by those evaluating the effects on human health. Regarding bioavailability, five studies tested the absorption and metabolism of GSLs from broccoli and broccoli sprouts. One study foresaw the administration of a single portion of broccoli (one serving or a dose equivalent to 200–300 g of raw product), while four studies a medium-long term intervention (range 14–64 days) and included the administration of two portions/day (at about 100 g each of broccoli) or three portions/week (300 ml each of broccoli soup). The main target tissues were plasma and urine followed by prostate tissue. Four out of five studies on GSL-rich foods were already published. Charron et al. (46) aimed to assess the change in GSL-metabolites measured in plasma and urine after the acute consumption of 100 g of broccoli and 10 g of daikon radish (providing about 100 μmol ITCs). The authors found that the absorption and metabolism of GSLs from cooked broccoli were widely affected by the body mass index (BMI) of the subjects. In particular, subjects with high BMI (i.e., higher than 26 kg/m^2^) had elevated levels of plasma and urinary metabolites and a delayed maximal plasma metabolite peak compared to those with low BMI (i.e., lower than 26 kg/m^2^). The authors attributed this BMI-associated response to the different gut transit times and/or differences in gut microbiota composition. Conversely, the same authors previously reported that subjects with high BMI showed higher plasma AUC and urinary excretion rates of total GSL and GSL-metabolites (erucin, SFN, and SFN-metabolites) following the consumption of control diet compared to the broccoli diet. Whereas, subjects with low BMI reported higher plasma AUC and urinary excretion rates following the administration of a broccoli diet (200 g of cooked broccoli and 20 g of raw daikon radish, providing 97.5 μmol of GR and 5.8 μmol of glucoerucin) for 15 days and followed by two single administrations the days after. In detail, the plasma AUC of total metabolites of low BMI subjects was 17% higher during the broccoli diet compared to the control diet (46). Sivapalan et al. (17) evaluated the absorption and excretion of GR, glucoerucin, and metabolites in plasma and urine of healthy subjects after the acute intake of 3 soups (300 g each) made from different broccoli genotypes (range of GR 84–452 μmol). The authors reported that the absorption and excretion of GSLs and metabolites were dependent on broccoli genotype and higher for the cultivar rich in GSLs. Finally, Coode-Bate and coworkers (49) reported no detectable levels of GSLs and derivatives in prostate biopsy tissues after 4-week consumption of three 300 ml portions/week of a broccoli soup (providing at about 280 μmol of GR and 1,513 μmol of (+)-S-methyl cysteine sulfoxide). On the contrary, the authors documented high levels of the sulfate metabolite (+)-S-methyl cysteine sulfoxide in the same target tissue and urine.

The main food sources used for studying the effects on human health were broccoli and broccoli sprout. These were tested both in healthy subjects and in those with risk factors. Only few studies were performed on subjects with prostate cancer. The duration of the intervention varied from a few days to until 10 weeks, while the dose of food was in the range of 30–200 g per day. The main markers considered included those related to inflammation, detoxification (mainly gene expression or enzymatic activity), and cancer risk analyzed mainly in the blood, targeted cells (peripheral blood mononuclear cells), or tissue (e.g., prostate). Noah et al. (39) showed that the ingestion of 200 g of BSH for 4 days was able to significantly reduce virus-induced markers of inflammation, such as IL-6, in smoker subjects compared to the placebo. Similarly, López-Chillón et al. (47) observed a significant decrease in plasma levels of IL-6 and C-reactive protein following 10-week consumption of broccoli sprouts (30 g/day) in healthy overweight subjects. Regarding the biomarkers of oxidative stress, Nguyen and coworkers (45) found a significant reduction of reactive oxygen species (ROS) and p38 MAP kinase in healthy non-smoker subjects following ingestion of 200 g of BSH compared to the placebo (alfalfa sprout homogenates). Doss et al. (40) demonstrated that the treatment for 21 days with 150 g of BSH in adults with sickle cell disease determined the activation of nuclear factor erythroid 2-related factor 2 (Nrf2) in erythroid progenitors and significantly increased the expression of Nrf2 targets such as heme oxygenase 1 (HO-1), NAD(P)H Quinone Dehydrogenase 1 (NQO1), and hemoglobin subunit gamma 1 (HBG1), restoring oxidative capacity. On the other hand, Sudini et al. (43) did not find any difference in transcript levels of Nrf2 target antioxidant genes HO-1, NQO1, glutamate cysteine ligase catalytic subunit (GCLC), and glutamate cysteine ligase modifier subunit (GCLM) in nasal epithelial cells and peripheral blood mononuclear cells (PBMCs) between the treatment with 100 g broccoli sprouts for three consecutive days and the placebo (alfalfa sprouts) among adults with asthma and allergic sensitization.

#### Characteristics and Results of the Studies Performed on GSL-Rich Extracts/Pure Compounds

The main characteristics of the studies performed on GSL-rich extracts/pure compounds are reported in Table 4. The bioavailability of GSLs and derivatives obtained from extracts and/or pure components have been evaluated in 13 studies. The main compounds tested were broccoli sprout extracts and SFN, analyzed in plasma and urine and in line with the studies performed on GSL-rich foods reported above. Some of the trials combined the study of bioavailability with that of the health effect. The dose administered was variable depending on the extract/compound tested (from 8 up to 600 μmol) and mainly in the form of pills or capsules. The duration of the studies was varying between one or few days up to 12 weeks depending on the aim of the research. The markers associated with the bioavailability included the dosage of the native GSL, derivatives (e.g., SFN), or metabolic products analyzed mainly in plasma and urine while those related to human health included markers directly and/or indirectly associated with prostate cancer, cognitive function, and cardiovascular health including inflammatory and antioxidant markers detected in plasma or target tissues. Only half of the studies have already been published. Regarding the bioavailability, Chen et al. (48) documented an increase in urinary SFN metabolites following the administration for 10 days of three different broccoli sprout beverages (100 ml each) containing different concentrations of GR (range 120–600 μmol) and SFN (range 8–40 μmol). In addition, the authors found a significant increase in benzene mercapturic acids in the urine of the group of subjects consuming the high-dose beverage. In another study (53), a 2-week intervention with 250 mg/day of a broccoli seed extract containing GR (224 mg/day) induced a significant change in total urinary SFN and individual SFN metabolites in a group of women scheduled for breast biopsy. Recently, Zhang et al. (61) reported a significant increase in total and individual SFN metabolites in urine and plasma following 2-week consumption of 2 capsules/day (providing 200 μmol of SFN in total) in a group of subjects scheduled for prostate biopsy.

Concerning the effects on human health with pure GSLs, Wise and coworkers (57) found no differences in Nrf2 target gene expression such as NQO1, HO1, aldo-keto reductase Family 1 Member C1 (AKR1C1), and aldo-keto reductase Family 1 Member C3 (AKR1C3) in alveolar macrophages and bronchial epithelial cells after intervention with 150 μmol SFN daily by mouth for 4 weeks in smoker subjects with physician diagnosed chronic obstructive pulmonary disease (COPD).

Regarding the effect of GSLs on cancer biomarkers, Atwell et al. (53) evaluated the role of SFN on PBMCs histone deacetylase (HDAC) activity, and tissue biomarkers (H3K18ac, H3K9ac, HDAC3, HDAC6, Ki-67, p21) in women with abnormal mammograms and scheduled for breast biopsy. After 2–8 weeks of supplementation with 250 mg of broccoli seed extract containing 30 mg of GR, the authors found a significant decrease in PBMC HDAC activity in the SFN group compared with the placebo group, while no significant effect was observed on examined tissue biomarkers. Conversely, in a total of 98 men scheduled for prostate biopsy, Zhang et al. (61) did not find significant differences in HDAC activity following the intervention with broccoli sprout extract (BSE) (200 μmol SFN) compared to the placebo. However, within the subgroup of subjects with a confirmed prostate cancer diagnosis, SFN showed a significant increase in HDAC activity.

Other prevalent biomarkers investigated were those related to cognitive function. A placebo-controlled, double-blind, randomized trial (52) showed the beneficial role of 50–150 μmol of SFN supplemented in young men (age 13–27) with moderate to severe autism spectrum disorder. The intervention with broccoli sprouts extract, rich in SFN, for 18 weeks was able to significantly improve behavioral measures such as the Aberrant Behavior Checklist (ABC), Social Responsiveness Scale (SRS), and Clinical Global Impression Improvement Scale (CGI-I) compared to the placebo group. Also Bent and coworkers (59) reported an improvement of specific symptoms of autism spectrum disorder in children and young adults following SFN supplementation. In particular, the study participants received daily SFN tablets (~2.5 μmol GR) for 12 weeks, and at the end of the study, both the ABC and the SRS parameters showed an improvement.

Although several biomarkers were positively modulated by the intervention with GSL-rich foods and GSL-rich extracts/pure compounds, different studies reported an apparent null effect on lipid profile and vascular function. For instance, a randomized, 3-arm parallel, controlled study (38) found no effect on total cholesterol (TC), systolic blood pressure (SBP), diastolic blood pressure (DBP), high-density lipoprotein cholesterol (HDL-C), low-density lipoprotein cholesterol (LDL-C), triglycerides (TG), oxidized LDL (ox-LDL), high-sensitivity C-reactive protein (hsCRP), pulse wave velocity (PWV), and augmentation index (Aix) following 12-week dietary intervention with 400 g of high-GR broccoli (21.6 ± 1.60 μmol/g dry-weight GR and 4.5 ± 0.34 μmol/g dry-weight glucoiberin) compared with standard broccoli (6.9 ± 0.44 μmol/g dry-weight GR and 0.7 ± 0.33 μmol/g dry-weight glucoiberin) or peas. Also, Christiansen et al. (37) did not observe an improvement in FMD as a marker of vascular function, as well as BP, HDL-C, and LDL-C in hypertensive individuals after 4-week period intervention with 10 g of dried broccoli sprouts daily (25.9 ± 8.5 μmol/g dry-weight GR; 48.5 ± 14.2 μmol/g dry-weight total GSLs) compared to the habitual diet. Similarly, Axelsson et al. (58) reported no significant effect of SFN (daily dose of 150 μmol for 12 weeks) on TC and TG compared with the placebo group in patients with type 2 diabetes.

## Discussion

The study of the role of GSLs and GSL-rich foods on human health has received increasing interest, as documented by the boost of human intervention trials carried out on this topic. Our review was focused on analyzing the trend of the registered studies on GSLs by using ClinicalTrials.gov and ISRCTN registry as databases. In this study, we summarized the main characteristics of the studies in order to provide a general and broad overview in the research field of GSLs to sum up what has already been done including the main gaps to be filled and, in turn, how and where to direct new efforts on this field.

### Studies Performed on GSL-Rich Foods

Regarding foods, numerous epidemiological studies have shown an inverse association between the consumption of GSL-rich foods (i.e., broccoli, kale, cabbage, and cauliflower) and the risk of different types of cancer, including colorectal, breast, and prostate cancer (27, 62, 63). In this study, we documented that most of the registered clinical trials were focused on broccoli, in line with the trials registered on the extracts, while very few dietary interventions were registered on other brassica vegetables. These studies included Brussel sprout, kale, mustard, and watercress (64–69). One possible explanation could be related to their limited human consumption and generally restricted to certain populations. However, the effects of these brassica vegetables on human health have been investigated in the past both through observational and intervention studies (70–72). The main topics included the evaluation of oxidative stress and cardiometabolic markers. These research themes have been partially confirmed in the current revision even if the actual research trend on these vegetables includes the evaluation of the metabolism of polycyclic aromatic hydrocarbons, the study of cell phase angle (as an indirect measure of the cell membrane integrity, function, and metabolism), the activity of phase I and II enzymes, and the evaluation of energy expenditure. Only one of the registered studies has been published at present. The results documented that the short-term consumption of 10 g/day of mustard (providing about 16 mg of allyl-ITC) failed to affect energy expenditure and metabolic markers (i.e., plasma glucose, fatty acids) in a group of non-smoker individuals (44).

Most of the registered and published trials that were analyzed have investigated the effects of broccoli and broccoli sprout on different health status parameters. The few results available could suggest a possible beneficial effect against the inflammation as documented through the reduction of some markers such as IL-6 and C-reactive protein in smokers and in overweight subjects following the consumption of broccoli sprout. Only one study reported an attenuation in the expression of genes and associated oncogenic pathways of prostate cancer following a 4-week intervention with 300 g of GR-rich broccoli soup (11).

Concerning the impact on gut microbiota, the literature on GSLs is very recent, but it seems to suggest a strict relationship between the consumption of GSLs, their metabolism, and the composition of gut microbiota (73). In the present review, only two registered studies were focused on the impact of GSLs on gut microbiota composition. One study was performed on broccoli and not published yet (74), while another study was performed by administering a diet rich in Brassica vegetables (75). The latter already published (76), documented the capacity of a diet rich in Brassica vegetables (consisting of six portions of 84 g of broccoli, six portions of 84 g of cauliflower, and six portions of 300 g of a broccoli and sweet potato soup each week, for a period of 2 weeks) to increase human gut lactobacilli and to reduce the abundance of sulfate-reducing bacteria compared to a low Brassica vegetable-diet. However, no information on GSLs absorption and gut metabolism has been reported by the authors. Data from an *in vitro* study documented the formation of amines from the secondary degradation of ITCs following the incubation of human feces with GSLs (77), while another study showed the potential *in vitro* role of Bifidobacterium strains (belonging to the human intestinal microbiota) to metabolize GSLs into nitriles (25).

### Studies Performed on GSL-Rich Extracts/Pure Compounds

As expected, we documented a rising trend of the last 10 years of studies performed on extracts or pure compounds despite only a few were those carried out on GSL-rich foods. This trend that is in favor of extracts and pure components, despite the difficulties in undertaking a human dietary intervention with a food product, probably derives from the growing interest of the pharmaceutical industry toward the potential use of GSLs, not only as food supplements but also as drugs. We have found that the research of the last years on GSL extracts was mainly focused on broccoli extracts and SFN (the main GSL-metabolite from broccoli). In this regard, several preclinical studies have been performed with the intention to reveal the mechanisms behind the protection against the development and/or progression of different diseases, particularly, cancer (78). The main putative protective mechanisms included the induction of endogenous antioxidants defense, detoxification enzymes, and the activation of cytoprotective genes (70, 79). Furthermore, in a recent review, it has been reported that SFN, indole-3-carbinol, and 3,3-diindolylmethane were the main GSL-metabolites showing a potential beneficial effect against diabetes, cancer, and neurodegenerative diseases (80). The biological effects observed were attributed to a plurality of molecular mechanisms acting simultaneously which included the modulation of xenobiotic metabolism, modulation of inflammation, regulation of apoptosis, cell cycle arrest, angiogenesis, and metastasis and regulation of epigenetic events (80, 81). The study of the potential health effects of SFN has a long-standing history showing its ability to act as an inducer of Nrf2. Several studies have also documented the ability of SFN to produce significant clinical responses in cancer and cognitive diseases (82–84). The main targets included the analysis of the expression of genes relevant for cellular protection and antioxidant activity such as Nrf2 in addition to epigenetic aspects such as the modulation of HDAC6 (82–84). These characteristics make SFN a potential candidate for the development of supplements and could explain its growing interest in the pharmacological research area. However, it should be underlined that most of the already published studies administered doses of ITCs from physiological to supraphysiological concentrations (range 100–600 μmol). For example, Duran et al. (41) found an increase in SFN conjugate levels in plasma following *in vivo* supplementation for 3 days with 200 g of BSH compared to placebo, but the final concentration of metabolites was in the order of magnitude of nanomolar. In fact, the authors supposed that the minimal effects on antioxidant gene expression observed were probably due to insufficient plasma levels of SFN achieved. Furthermore, levels achieved in target tissues were likely less than those achieved in the plasma. In contrast, in another study, supplementation with 200 μmol of SFN equivalents (from BSE) determined plasma levels of SFN metabolites at a magnitude of μmol after 3 h and was able to elicit HDAC inhibitory responses *in vivo* (54). This discrepancy in the results obtained could be attributed to the different experimental designs in terms of extract administered, doses, and duration of intervention. Apart from the studies investigating the bioavailability of GSLs and their metabolites, the rest of the trials were focused on the role of the immune system, inflammatory response, cognitive function, cancer, and metabolic parameters (e.g., blood glucose). The results were not univocal and strictly dependent on the marker analyzed, site of measurement, dose administrated, and duration of the intervention. The preliminary findings obtained from human intervention studies have documented a possible beneficial effect from the consumption of broccoli sprout extract containing SFN (generally for doses close to 200 μmol) in the expression of genes related to cancer (53, 61) and in the modulation of markers of inflammation (51). However, due to the limited number of studies published, further investigations are mandatory before drawing any conclusion.

### Effect of Interindividual Response on GSLs Metabolism

Another important aspect considered in the metabolism of GSLs is the impact of the genetic glutathione S-transferase (GST) polymorphisms that could affect interindividual response. For instance, Gasper et al. (85) demonstrated a relevant role of the GSTM1 allele in the metabolism of dietary ITCs. The authors found that GSTM1-null and GSTM1-positive subjects have different SFN metabolites concentrations in plasma and different rates of urinary excretion of SFN metabolites after broccoli consumption that could be explained by the GSTM genotype. These results were in line with the observations reported by other authors documenting a different GST activity and metabolism of GSLs (86, 87). Furthermore, numerous epidemiological studies associated GST polymorphisms with a different level of protection against oxidative stress other than an increased susceptibility to cancer diseases (88, 89). The study of the impact and/or contribution of GST polymorphic genes on GSLs bioavailability and/or health related outcomes has been considered only in two registered clinical trials but not published yet. One study aimed to investigate the impact of broccoli consumption (400 g of the high GSLs broccoli each week for 12 weeks) on CVD risk by considering the potential contribution of the different polymorphisms (GSTM1, GSTT1, and GSTP1) (90). The second study aimed to determine the possible differential effects of the GSTM1 genotype, GSTT1 genotype, and their combined effects on PEITC-NNK association on the metabolism and excretion of PEITC (91). The results will be useful to provide more data on the potential impact and/or contribution of GST polymorphisms and genotypes on the capacity to differently metabolize GSLs. In addition, the results obtained on CVD risk will be pivotal for further research on this relatively new Research Topic.

### Current Limitations and Gaps of the Research

Overall, the main limitations and criticisms that emerged from the analysis of the studies include the following: (1) a lower number of subjects were enrolled and their heterogeneous characteristics, (2) the high variability of the individual response, (3) the poor quality of the study design adopted (e.g., some studies lack a control group), (4) the amount of food administered that in some cases resulted below the threshold reported in epidemiological studies and in some cases the amount of extracts and/or single compounds provided that was above to the dose achievable through the diet, and (5) the lack of validated biomarkers. In this regard, a strong gap of the research remains the validation of appropriate biomarkers directly linked to the endpoint and able to be affected following a dietary intervention.

A further gap is insufficient information related to the absorption and metabolism of GSLs and their metabolites from single and different food sources. This could be partially attributed to the complex metabolism and/or to the absence of standards and methodologies for the identification of the compounds. Since GSLs are extensively metabolized, the identification of more specific dietary biomarkers able to trace their metabolism represents a challenging achievement. Another important gap that should be addressed is the study of the potential interaction among different GSLs and the interaction between GSLs with other dietary components present in the brassica or in the whole diet. In fact, it is important to point out that food should be tested in its pattern of consumption within a context of a balanced, adequate, and varied diet in which the contribution of the single food or food component should be considered. In this regard, many dietary and non-dietary factors could positively or negatively influence the digestion and absorption of GSLs/GSL-metabolites, their excretion, and tissue distribution. Furthermore, it cannot be excluded a possible additive or synergistic or antagonist effect when GSLs are administered together with other dietary or non-dietary factors. Another relevant point is that the lack of exhaustive information is related to the degradation rate of GSLs during food processing and the potential impact of their bioavailability. Despite numerous studies performed, a major effort should be directed on this topic by combining, for example, the use of predictive models able to study such phenomena and capable to predict the bioavailability *in vivo*. In addition, the impact of age, sex, microbiome composition, and genetic polymorphisms on absorption, distribution, metabolism, and excretion of GSLs should be deeply investigated. In part, this aspect has been already analyzed within the POSITIVE project also for GSLs (92). However, the study of the individual response is crucial for a clear understanding of the impact of GSLs and derivatives on human health.

### Strength and Limitations of the Revision

Overall, the following review presents several strengths and limitations. One strength is the capacity to provide a prompt and updated list of clinical trials performed on GSLs including those that are not yet published. Additionally, the registered studies can provide information that is often missing such as specific details on the characteristics of the study population, study design, and all outcomes measured. On the other hand, several limitations can be remarked. For example, a relevant limit is represented by the incomplete real overview of the number of studies performed on GSLs and GSL-rich foods due to the lack of their registration in a public registry. This problem is tangible particularly for the studies performed in the past since registration was not considered common practice. Another important limitation is the lack of information related to the results of the studies. Most of them are very recent and not already published, and this does not allow to have a clear overview of all the results.

## Conclusions and Future Directions

In conclusion, GSLs and GSL-rich foods have attracted great interest in the research field of dietary bioactives. Most of the human intervention studies were performed on SFN and broccoli. Apart from the study of GSLs metabolism, other Research Topics included cancer, oxidative stress, and inflammation. The results available remain conflicting and further effort should be done to clarify the role of pure/extracts and foods on these matters. However, new Research Topics have been developed including the study of gut microbiota and its contribution to GSLs metabolism, and the impact of GSLs and GSL-rich foods on skin health and cognitive function. It seems important that future research consider the contribution of the single components and/or foods, the doses administered, and, above all, the individual response in terms of gut microbiota and genetic polymorphisms that significantly affect the absorption, along with the metabolism of GSLs. For this purpose, the development of dietary intervention studies is highly recommended by taking into consideration also the dose-response behavior and the mechanisms of interaction between the GSLs/GSL-metabolites and their target tissues. For this latter point, the use of validated biomarkers is mandatory. It would be also interesting to enlarge the research to different and less common food sources of GSLs (e.g., kale, rocket salad, and mustard) since they are distinctive of peculiar secondary GSL metabolites whose potential bioactivity has not been evaluated yet. Moreover, the increased knowledge on the contribution of the different classes and types of vegetables in terms of bioactive compounds and the evaluation of their modulatory role represents an important aspect in the context of a plant-based diet.

## Author Contributions

MM and CDB conducted the literature search and reviewed the studies selected. DM served as the third reviewer. MM, SV, and MT prepared the tables and figures. MM, DM, and CDB were involved in the protocol design, data analyses, and drafted the manuscript. PR and MP critically revised the scientific content and improved the quality of the manuscript. All authors contributed to the article and approved the submitted version.

## Funding

This research is part of the project MIND FoodS HUB (Milano Innovation District Food System Hub): Innovative concept for the eco-intensification of agricultural production and for the promotion of dietary patterns for human health and longevity through the creation in MIND of a digital Food System Hub, co-funded by POR FESR 2014-2020_BANDO Call HUB Ricerca e Innovazione, Regione Lombardia. Open access publication fees will be provided by Piano di sostegno alla ricerca- Linea 2, azione A-Grant Nos. PSR2020-CDELB.

## Conflict of Interest

The authors declare that the research was conducted in the absence of any commercial or financial relationships that could be construed as a potential conflict of interest.

## Publisher's Note

All claims expressed in this article are solely those of the authors and do not necessarily represent those of their affiliated organizations, or those of the publisher, the editors and the reviewers. Any product that may be evaluated in this article, or claim that may be made by its manufacturer, is not guaranteed or endorsed by the publisher.
